# Comparative Transcriptional Profiling of Melatonin Synthesis and Catabolic Genes Indicates the Possible Role of Melatonin in Developmental and Stress Responses in Rice

**DOI:** 10.3389/fpls.2016.00676

**Published:** 2016-05-18

**Authors:** Yunxie Wei, Hongqiu Zeng, Wei Hu, Lanzhen Chen, Chaozu He, Haitao Shi

**Affiliations:** ^1^Hainan Key Laboratory for Sustainable Utilization of Tropical Bioresources, College of Agriculture, Hainan UniversityHaikou, China; ^2^Key Laboratory of Biology and Genetic Resources of Tropical Crops, Institute of Tropical Bioscience and Biotechnology, Chinese Academy of Tropical Agricultural SciencesHaikou, China; ^3^Institute of Apicultural Research, Chinese Academy of Agricultural SciencesBeijing, China

**Keywords:** melatonin, rice, gene expression, circadian rhythm, development, immunity, stress response

## Abstract

As a well-known animal hormone, melatonin (*N*-acetyl-5-methoxytryptamine) is also involved in multiple plant biological processes, especially in various stress responses. Rice is one of the most important crops, and melatonin is taken in by many people everyday from rice. However, the transcriptional profiling of melatonin-related genes in rice is largely unknown. In this study, the expression patterns of 11 melatonin related genes in rice in different periods, tissues, in response to different treatments were synthetically analyzed using published microarray data. These results suggest that the melatonin-related genes may play important and dual roles in rice developmental stages. We highlight the commonly regulation of rice melatonin-related genes by abscisic acid (ABA), jasmonic acid (JA), various abiotic stresses and pathogen infection, indicating the possible role of these genes in multiple stress responses and underlying crosstalks of plant hormones, especially ABA and JA. Taken together, this study may provide insight into the association among melatonin biosynthesis and catabolic pathway, plant development and stress responses in rice. The profile analysis identified candidate genes for further functional characterization in circadian rhythm and specific stress responses.

## Introduction

Melatonin (*N*-acetyl-5-methoxytryptamine) was first discovered in the cow’s pineal gland (Lerner et al., 1958). Dubbels et al. (1995) and Hattori et al. (1995), melatonin was identified in plants by two research groups. Until now, melatonin has been found in multiple plant species, including alfalfa, almond, anise, apples, *Arabidopsis*, banana, beetroot, bermudagrass, black mustard, cabbage, celery, cherry, coriander, cucumber, fennel, fenugreek, flax, green cardamom, milk thistle, oranges, poppy, potato, rice, sunflower, tobacco, tomato, white mustard, wolf berry, etc. (Manchester et al., 2000; Zhao et al., 2013; Shi and Chan, 2014). In addition, the endogenous melatonin concentration can also be modulated through genetic transformation in tomato and rice (Okazaki and Ezura, 2009; Okazaki et al., 2009, 2010; Byeon et al., 2012, 2013, 2014; Byeon and Back, 2014a,b).

**FIGURE 1:**
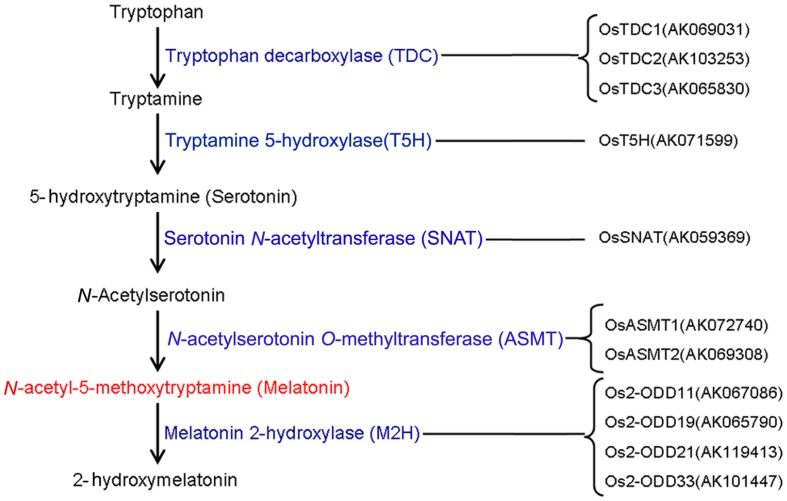
**The genes responsible for melatonin synthesis from tryptophan and melatonin degradation in rice**.

To date, the biosynthesis and metabolic pathways of melatonin in plants have been established (**Figure 1**). Melatonin in plants can be synthesized by four sequential enzymes from tryptophan (Kang et al., 2011), including TDC, T5H, SNAT, and *N*-aceylserotonin *O*-methyltransferase (ASMT) (Arnao and Hernández-Ruiz, 2014, 2015; Zuo et al., 2014). Thereafter, melatonin is catabolized by M2H into 2-hydroxymelatonin (Byeon and Back, 2015). In rice, gene families of TDC, T5H, SNAT, and ASMT contain 3, 1, 1, and 3 known members, respectively (Kang et al., 2007; Fujiwara et al., 2010; Kang et al., 2013; Park et al., 2013a). However, *OsASMT3* is barely detectable in any of the plant organs (Park et al., 2013b). *OsM2H* genes belong to 2-ODD family and at least 4 of 2-ODD genes show M2H activities in rice (Byeon and Back, 2015).

Solid evidence implicates that melatonin is involved in multiple plant biological processes and various stress responses (Hardeland, 2015; Zhan et al., 2015), including circadian rhythm (Kolár and Machácková, 2005; Arnao and Hernández-Ruiz, 2015), delayed senescence of leaves (Byeon et al., 2012; Wang et al., 2012, 2013a,b), leaf morphology (Okazaki et al., 2010), root development (Hernández-Ruiz et al., 2005; Pelagio-Flores et al., 2012; Zhang N. et al., 2014), coleoptile growth (Hernández-Ruiz et al., 2004, 2005), grain yield (Byeon and Back, 2014a), fruit ripening (Sun et al., 2015), drought stress (Wang et al., 2013a, 2014; Zhang et al., 2013; Meng et al., 2014; Zuo et al., 2014; Shi et al., 2015b), salt stress (Wei et al., 2014; Zhang H.J. et al., 2014; Liang et al., 2015; Shi et al., 2015b), cold stress (Posmyk et al., 2009a; Arnao and Hernández-Ruiz, 2014; Bajwa et al., 2014; Shi and Chan, 2014; Turk et al., 2014; Shi et al., 2015b), high temperature (Tiryaki and Keles, 2012), copper stress (Posmyk et al., 2008, 2009b), oxidative stress (Park et al., 2013b; Shi et al., 2015d), cadmium stress (Byeon et al., 2015) and pathogen infection (Yin et al., 2013; Lee et al., 2014, 2015; Reiter et al., 2015; Shi et al., 2015a; Zhao et al., 2015).

Melatonin plays protective roles in the regulation of plant tolerance to abiotic stress and biotic stress (Yin et al., 2013; Lee et al., 2014, 2015; Zhan et al., 2015). Overexpression of *OsTDC* increases endogenous melatonin level and delays leaf senescence in rice (Kang et al., 2007, 2009; Byeon et al., 2014). The transcript of *OsT5H* can be induced by *Magnaporthe grisea* infection (Fujiwara et al., 2010). Exogenous application of serotonin, the penultimate substrate for melatonin biosynthesis, induces defense gene expression and increases resistance to rice blast infection (Fujiwara et al., 2010). Transgenic rice plants ectopically expressing the AANAT regulates cold stress resistance (Kang et al., 2010), seminal root elongation (Park and Back, 2012), oxidative stress resistance (Park et al., 2013b), and seedling growth (Byeon and Back, 2014a). The transcript of *OsASMT* can be induced by ABA and methyl JA treatments, and *OsASMT* overexpressing plants result in higher level of melatonin (Park et al., 2013b). Exogenous application of melatonin improved apple resistance to Marssonina apple blotch (*Diplocarpon mali*) (Yin et al., 2013), enhanced disease defense against *Pseudomonas syringae* DC3000 in *Arabidopsis* and tobacco (Lee et al., 2014; Shi et al., 2015a).

Rice is one of the most important crops around the world, and melatonin is also taken in from rice by many people everyday. Thus, it is very useful and important to dissect the distribution and regulation of endogenous melatonin in rice. Melatonin is widely involved in plant development, multiple abiotic and biotic stress responses in *Arabidopsis* (Shi and Chan, 2014), and Bermudagrass (Shi et al., 2015b). However, transcriptional profiling of rice melatonin synthesis and catabolic genes has not been systematically carried out. In this study, we analyzed the expression profiling of 11 rice melatonin synthesis and catabolic genes in development, various tissues, and in response to hormone, pathogen infection, drought, salt, and cold stresses. These results may provide insight into the link among melatonin biosynthesis and catabolic pathway, plant development and stress responses in rice. Further functional characterization of identified candidate genes with potential involvement in circadian rhythm and stress responses through overexpressing, knocking down or knocking out will give more clues to melatonin-mediated signaling as well as underlying molecular mechanism.

## Materials and Methods

### Plant materials and Growth Conditions

Rice (*Oryza sativa* L. ssp. *japonica* cv. Nipponbare) seeds were sown in germinating boxes. At 30 days after germination, the seedlings were transplanted in a paddy field under normal conditions of the cultivation season. Thereafter, 56 DAT, 58 DAT, and 90 DAT were considered as the stage of panicle initiation, the early stage of panicle development indicating a complete reproductive transition, the stages of flowering and early stages of seed development corresponding to the ripening-stage transition, respectively.

For hormone treatments, rice seeds were germinated, and grown hydroponically in a growth chamber at 28°C under continuous light. Seven-day old seedlings were transferred in culture solution containing 50 μM ABA, or 10 μM GA, or 10 μM IAA, or 1 μM brassinolide (BL), or 1 μM tZ, or 100 μM JA, or in culture solution without hormone to serve as control (mock treatment). Samples were collected after 0, 0.25, 0.5, 1, 3, and 6 h incubation for root, and after 0, 1, 3, 6, and 12 h incubation for shoot.

### Pathogen Infection

Rice (*O. sativa* cv Nipponbare) plants grown in the greenhouse for 42 days were inoculated with *Xoo* T7174R, a wild-type strain, and 74HrcV::Km, a T3S-defective mutant by the leaf-clipping method. Plants treated with water were used as control. Leaf sections (3–5 mm) that included the inoculation site were collected at 3, 6, and 12 hpi and 1, 2, 4, 6 dpi.

For the blast fungus infection, three lines of rice cultivar Nipponbare carrying the blast resistance genes (*Pia*, *Pish*) were inoculated with two strains of *Magnaporthe oryzae* harboring *AVR-Pia* and *AVR-Pish*. Rice seedlings at the 4-leaf stage were placed in moist chamber and sprayed with a conidial suspension of *M. oryzae* (1 × 10^6^ conidia/ml). The seedlings were incubated in a moist chamber at 25°C for 24 h under dark condition, then grown in hydroponic culture under 14 h light (28°C) and 10 h dark (24°C). Leaf samples (4th leaf) from 3 individual experiments were harvested at 1, 2, 3, and 5 days post inoculation (dpi). Rice seedlings sprayed with water were used as control.

### Development- and Pathogen Infection-Related Data Analysis

The data of spatio-temporal transcript levels in various tissues or organs (RXP_0001), leaf and root transcriptional profile in light (RXP_003 and RXP_007) and dark (RXP_004 and RXP_008) throughout entire growth in the field, diurnal, and circadian leaf (RXP_002) and root (RXP_009) transcriptional profile throughout entire growth, plant hormone profile (RXP_001 to RXP_012), *Xoo*-treated profile (RXP_3002), and *M. oryzae*-treated profile (RXP_3001) were downloaded from RiceXPro^1^ (Sato et al., 2011a,b, 2013). All samples were used for hybridization using the Agilent one-color (Cy3) microarray-based gene analysis system. As detailed described in Sato et al. (2013), all the above data were deposited in GEO through the following accession numbers: GSE21396, GSE21397, GSE36040, GSE36042, GSE36043, GSE36044, GSE39423, GSE39424, GSE39425, GSE39426, GSE39427, GSE39429, and GSE39432. All the raw data were downloaded and re-analyzed for cluster analysis of expression profile that shown as normalized data (log_2_).

### Abiotic Stress-Related Data Analysis

As described in Jain et al. (2007), 7-day-old light-grown rice seedlings were transferred to control condition and 200 mM NaCl solution as salt stress for 3 h, were dried on tissue paper as dehydration stress for 3 h, and were kept at 4°C as cold stress for 3 h. Then the seedlings were sampled in triplicate. GEO series accession no. GPL2025 were used for microarray analysis as Jain et al. (2007) described. All the normalized data were obtained from Rice eFP Browser^2^ (Jain et al., 2007).

### Cluster Analysis

The original data from RiceXPro and Rice eFP Browser were listed in **Supplementary Table S1**. Hierarchical cluster analysis of transcriptional profile was performed using CLUSTER program^3^ (Larkin et al., 2007), and the heatmap was obtained using Java Treeview^4^ (Saldanha, 2004) according to the instructions.

## Results

### The Spatio-Temporal Transcript Levels of Rice Melatonin Synthesis and Catabolic Genes in Various Tissues or Organs

**FIGURE 2:**
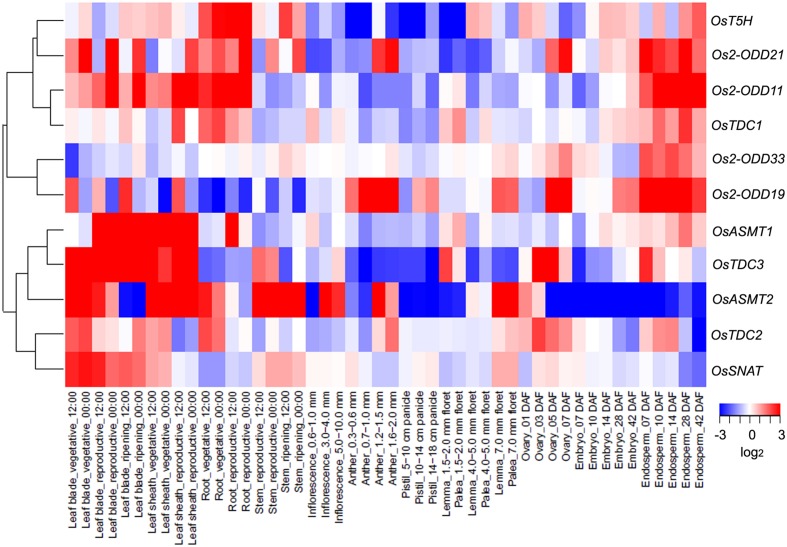
**The spatio-temporal transcript levels of rice melatonin synthesis and catabolic genes in various tissues or organs.** The transcriptional expression data were obtained from The Rice Expression Profile Database (RiceXPro) (http://ricexpro.dna.affrc.go.jp/). Rice (*O. sativa* L. ssp. *japonica* cv. Nipponbare) seeds were sown in germinating boxes. At 30 days after germination, the seedlings were transplanted in a paddy field under normal conditions during the cultivation season. Thereafter, 56 DAT, 58 DAT and 90 DAT were considered as the stage of panicle initiation, the early stage of panicle development indicating a complete reproductive transition, the stages of flowering and early stages of seed development corresponding to the ripening-stage transition, respectively. In various tissues or organs at different stages, a total of 48 samples in three replicates were used for hybridization using the Agilent one-color (Cy3) microarray-based gene analysis system. The cluster analysis of expression profile for each gene in various tissues is shown as normalized data (log_2_).

To investigate the expression profiles of rice melatonin synthesis and catabolic genes in various tissues or organs, we analyzed the expression of these genes using published microarray data (Sato et al., 2013). Eleven of rice melatonin-related genes have the corresponding probe sets in the dataset (**Figure 1**). As shown in **Figure 2**, all genes showed different expression pattern in various tissues, indicating that these genes may play different roles in plant growth and development. Interestingly, the expression patterns of melatonin-related genes could be divided into two groups (**Figure 2**). One group contained six genes (*OsT5H*, *Os2-ODD11*, -*19*, -*21*,-*33*, and *OsTDC1*), and most of them showed high expression levels in endosperm tissues. The other group contained five genes (*OsASMT1*, -*2*, *OsTDC2*, -*3*, and *OsSNAT*), and most of them showed high expression levels in leaf blade and leaf sheath tissues. Moreover, *Os2-ODD19* showed high expression level in four time points of anther, while other four genes (*OsTDC1*, -*3*, *Os2-ODD11*, and *OsASMT1*) exhibited lower expression level. Similarly, six genes (*Os2-ODD11*, -*19*, -*21*,-*33*, *OsTDC1*, and *OsASMT1*) showed high expression level in five time points of endosperm, while two genes (*OsASMT2* and *OsSNAT*) with a relative low level of expression. These melatonin-related genes showed high expression levels in a special tissue indicated their possible roles of melatonin in special tissue.

Moreover, three genes (*Os2-ODD19*, -*21*, and *OsTDC2*) showed different expression level in day and night at 9 tissues (leaf blade-vegetative, leaf blade-reproductive, leaf blade-ripening, leaf sheath-vegetative, leaf sheath-reproductive, root-vegetative, root-reproductive, stem-reproductive, and stem-ripening). The results indicate that *Os2-ODD19*, -*21*, and *OsTDC2* may play some roles in circadian rhythm and may be used in further functional characterization.

### Transcriptional Profile of Rice Melatonin Synthesis and Catabolic Genes throughout Entire Growth in the Field

**FIGURE 3:**
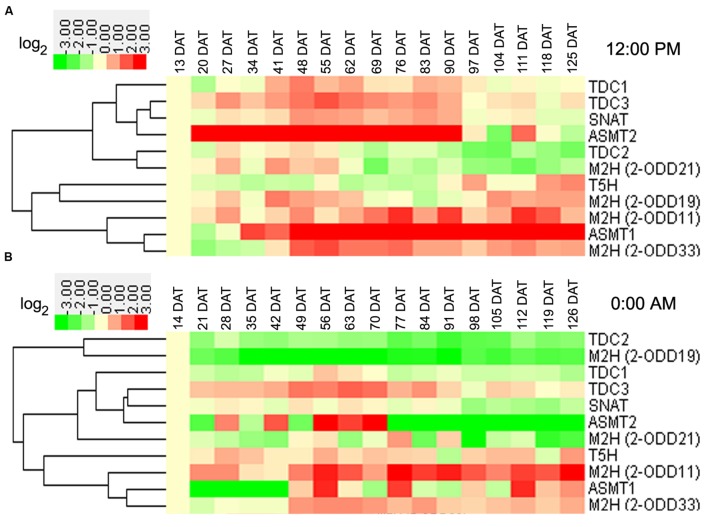
**Leaf transcriptional profile of rice melatonin synthesis and catabolic genes in light (A) and dark (B) throughout entire growth in the field.** The transcriptional expression data were obtained from RiceXPro (http://ricexpro.dna.affrc.go.jp/). Rice (*Oryza sativa* L. ssp. *japonica* cv. Nipponbare) seeds were sown in germinating boxes. At 30 days after germination, the seedlings were transplanted in a paddy field under normal conditions during the cultivation season. Samples corresponding to the uppermost fully expanded leaves were collected every 12:00 PM at weekly intervals from 13 to 125 DAT and every 0:00 AM at weekly intervals from 14 to 126 DAT. A total of 51 samples at 12:00 PM in three replicates and 34 samples at 0:00 AM in two replicates were used for hybridization using the Agilent one-color (Cy3) microarray-based gene analysis system. The cluster analysis of expression profile for each gene in various tissues is shown as normalized data (log_2_).

In rice leaves at day, the transcript levels of *OsTDC3* and *OsASMT2* were increased at vegetative stages, while that of *OsT5H* was decreased (**Figure 3A**). At reproductive stages, the transcripts of seven genes (*OsTDC1*, -*3*, *OsASMT1*, -*2*, *Os2-ODD11*, -*33*, and *OsSNAT*) and two genes (*OsTDC2* and *Os2-ODD21*) showed up-regulation and down-regulation, respectively (**Figure 3A**). At ripening stages, the transcripts of three genes (*Os2-ODD11*, -*33*, and *OsASMT1*) and two genes (*OsTDC2* and *Os2-ODD21*) showed up-regulation and down-regulation, respectively (**Figure 3A**). In the leaves at night, the transcript of *Os2-ODD11* showed up-regulation at all time points, while the transcripts of *OsTDC2* and *Os2-ODD19* were obviously down-regulated (**Figure 3B**). At vegetative stages, the transcripts of three genes (*OsTDC3*, *OsT5H*, and *Os2-ODD11*) and two genes (*OsTDC2* and *Os2-ODD19*) showed up-regulation and down-regulation, respectively (**Figure 3B**). At reproductive stages, the transcripts of four genes (*OsTDC3*, *OsT5H*, *Os2-ODD11*,-*33*) and two genes (*OsTDC2* and *Os2-ODD19*) showed up-regulation and down-regulation, respectively (**Figure 3B**). At ripening stages, the transcripts of two genes (*Os2-ODD11*, -*33*) and five genes (*OsTDC1*, -*2*, *Os2-ODD19*, *OsSNAT*, and *OsASMT2*) showed up-regulation and down-regulation, respectively (**Figure 3B**).

**FIGURE 4:**
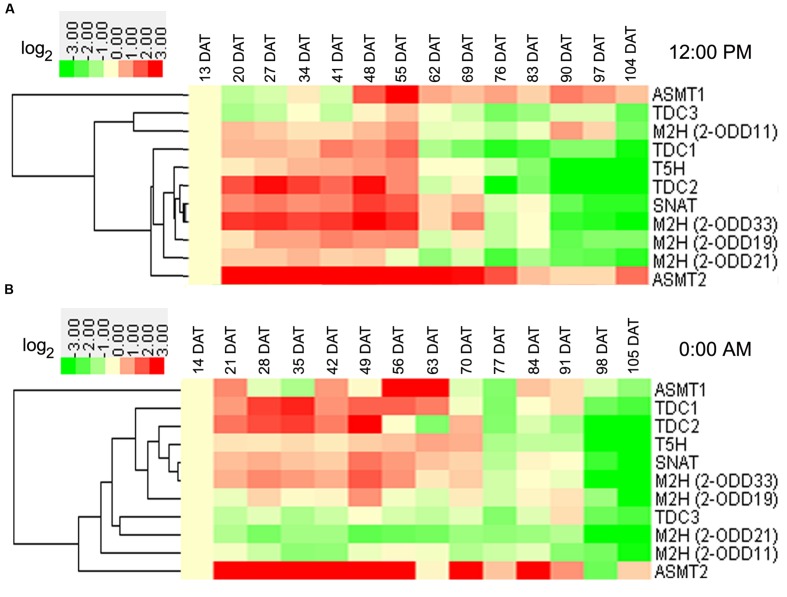
**Root transcriptional profile of rice melatonin synthesis and catabolic genes in light (A) and dark (B) throughout entire growth in the field.** The transcriptional expression data were obtained from RiceXPro (http://ricexpro.dna.affrc.go.jp/). Rice (*O. sativa* L. ssp. *japonica* cv. Nipponbare) seeds were sown in germinating boxes. At 30 days after germination, the seedlings were transplanted in a paddy field under normal conditions during the cultivation season. Root samples at various growth stages encompassing the vegetative, reproductive, and ripening stages were collected every 12:00 PM at weekly intervals from 13 to 104 DAT and every 0:00 AM at weekly intervals from 14 to 105 DAT. The cluster analysis of expression profile for each gene in various tissues is shown as normalized data (log_2_).

In rice roots at day, transcript of *OsASMT2* increased in the entire growth stages in the field (**Figure 4A**). At vegetative stages, the transcripts of eight genes (*Os2-ODD11*, -*19*,-*33*, *OsTDC1*, -*2*, *OsT5H*, *OsSNAT*, and *OsASMT2*) showed up-regulation (**Figure 4A**). At reproductive stages, the transcripts of two genes (*OsASMT1*, -*2*) and four genes (*OsTDC1*, -*3, Os2-ODD11*,-*21*) showed up-regulation and down-regulation, respectively (**Figure 4A**). At ripening stages, the transcripts of two genes (*OsASMT1* and -*2*) and eight genes (*OsTDC1*, -*2*,-*3*, *Os2-ODD19*, -*21*,-*33*, *OsT5H* and *OsSNAT*) showed up-regulation and down-regulation, respectively (**Figure 4A**). Interestingly, the transcripts of six genes (*OsTDC1*, -*2*, *Os2-ODD19*,-*33*, *OsT5H*, and *OsSNAT*) were obviously up-regulated at vegetative stages, but down-regulated at ripening stages (**Figure 4A**). In the roots at night, transcript of *Os2-ODD21* decreased throughout entire growth stages in the field (**Figure 4B**). At vegetative stages, the transcripts of six genes (*OsTDC1*, -*2*, *OsT5H*, *OsSNAT*, *Os2-ODD33*, and *OsASMT2*) and two genes (*Os2-ODD11*, -*21*) showed up-regulation and down-regulation, respectively (**Figure 4B**). Additionally, the transcripts of two genes (*OsTDC3* and *Os2-ODD21*) and five genes (*Os2-ODD11*, -*21*,-*33, OsTDC2*, and *OsT5H*) showed down-regulation at reproductive and ripening stages, respectively (**Figure 4B**). The transcripts of three genes (*OsTDC2*, *OsT5H*, and *Os2-ODD33*) showed up-regulation at vegetative stages, but down-regulation at ripening stages (**Figure 4B**).

Some genes shared similar expression patterns at day and night at the same stage (**Figures 3** and **4**). The transcripts of *Os2-ODD11* and *-33* showed up-regulation at reproductive and ripening stages in leaves, the transcripts of six genes (*OsTDC1*, -*2*, *OsT5H*, *OsSNAT*, *OsASMT2*, and *Os2-ODD33*) showed up-regulation at vegetative stages in roots, the transcripts of *OsTDC1* and *Os2-ODD21* shared down-regulation at reproductive and ripening stages of roots. However, there were also some genes shared different expression patterns. For example, the transcript of *OsT5H* shared down-regulation in day at vegetative stages in leaves, but showed up-regulation in night. Moreover, some genes also shared similar expression patterns in different tissues at the same stage. At day, the transcripts of *OsASMT2* and *OsASMT1* showed up-regulation at vegetative and ripening stages in leaves and roots, respectively, while the transcript of *Os2-ODD21* shared down-regulation at reproductive and ripening stages. On the contrary, some genes shared different expression patterns in different tissues at the same stage. The transcripts of *OsT5H* showed down-regulation in day at vegetative stages in leaves, but showed up-regulation in roots. The transcripts of *Os2-ODD11* and *Os2-ODD33* showed up-regulation at ripening stages of leaves, but showed down-regulation at roots. These results suggest that the melatonin-related genes as well as endogenous melatonin may play important and dual roles in rice developmental stages.

### Diurnal and Circadian Transcriptional Profile of Rice Melatonin Synthesis and Catabolic Genes throughout Entire Growth

**FIGURE 5:**
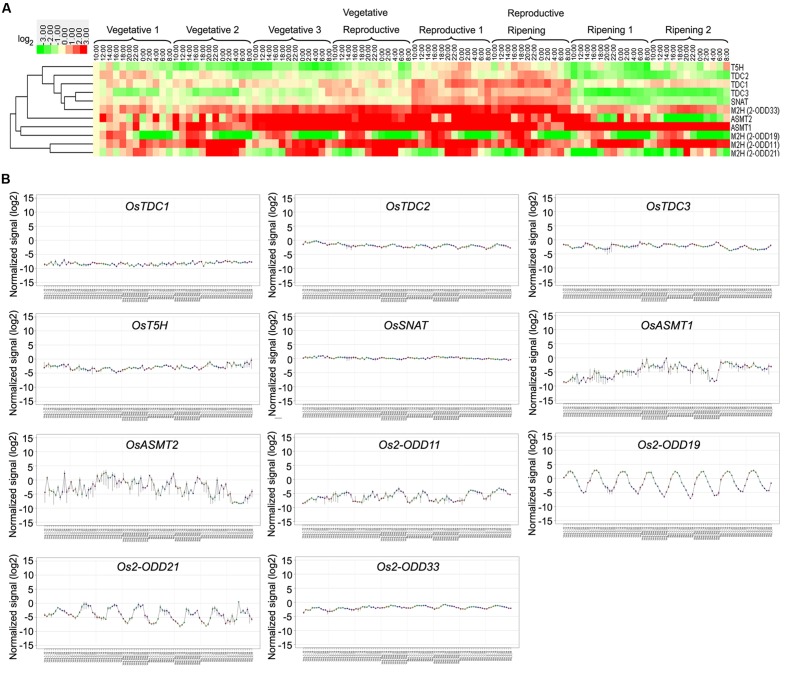
**Diurnal and circadian leaf transcriptional profile of rice melatonin synthesis and catabolic genes throughout entire growth as shown by heatmap (A) and line chart (B).** The transcriptional expression data were obtained from RiceXPro (http://ricexpro.dna.affrc.go.jp/). Rice (*O. sativa* L. ssp. *japonica* cv. Nipponbare) seeds were sown in germinating boxes. Gene expression profile of rice plants grown under natural field conditions based on microarray analysis of leaf samples at various growth stages encompassing the vegetative, reproductive and ripening stages. Samples corresponding to the uppermost fully expanded leaves were collected in a 48-h period at 2-h intervals at eight different growth stages. The cluster analysis and normalized signal of expression profile for each gene in various tissues is shown as normalized data (log_2_).

As shown in **Figure 5A**, *Os2-ODD11* expression was induced throughout entire growth stages, and the transcripts of four genes (*OsASMT1*,-*2 OsTDC1* and *Os2-ODD33*) were induced at most time points of growth stages, while those of *Os2-ODD19* and *Os2-ODD21* were intermittent. Before reproductive 1 stage, *Os2-ODD21* showed significant induction at night. On the contrary, *Os2-ODD19* was obviously down-regulated. This result suggested that *Os2-ODD19* and *Os2-ODD21* may play dual and important roles in the regulation of circadian rhythm. Moreover, the transcripts of four genes (*Os2-ODD11*, -*33*, *OsASMT1*, and -*2*) and two genes (*OsASMT1* and *Os2-ODD11*) displayed significant up-regulation at four stages (vegetative 3, vegetative-reproductive, reproductive 1, and reproductive-ripening stages) and two stages (ripening 1 and 2 stages), respectively. However, *OsTDC2*, *OsTDC3*, and *OsSNAT* expressions were repressed during ripening 1 and 2 stages. Additionally, the transcripts of most genes were induced during reproductive 1 and reproductive-ripening stages. At the last two stages (ripening 1 and 2 stages), most of genes were obviously down-regulated. Interestingly, the transcripts of five genes (*OsTDC2*, -*3*, *OsT5H*, *OsSNAT*, and *OsASMT2*) were induced during reproductive 1 and reproductive-ripening stages, but exhibited down-regulation at ripening 1 and 2 stages. As shown in **Figure 5B**, the expression of *Os2-ODD19* and *Os2-ODD21* displayed obviously regular change throughout entire growth.

**FIGURE 6:**
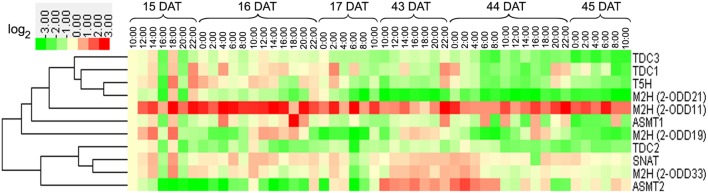
**Diurnal and circadian root transcriptional profile of rice melatonin synthesis and catabolic genes throughout entire growth.** The transcriptional expression data were obtained from RiceXPro (http://ricexpro.dna.affrc.go.jp/). Rice (*O. sativa* L. ssp. *japonica* cv. Nipponbare) seeds were sown in germinating boxes. Root samples corresponding were collected in a 48-h period at 2-h intervals at two different growth stages. The cluster analysis of expression profile for each gene in various tissues is shown as normalized data (log_2_).

In roots, the transcript of *Os2-ODD11* was up-regulated at all time-points, while those of *OsTDC3*, *Os2-ODD21*, and *OsTDC2* were down-regulated (**Figure 6**). The transcriptional profile of *Os2-ODD19* was intermittent, which was consistent with the result in leaves. During 15–17 DAT, the expressions of *Os2-ODD21*, *OsTDC2*, and *OsASMT2* were repressed. The transcript of *Os2-ODD11* was significantly induced during 43–45 DAT, whereas the transcripts of *OsTDC1*, -*2*, -*3*, *OsT5H*, and *Os2-ODD21* were repressed.

### The Transcriptional Profile of Rice Melatonin Synthesis and Catabolic Genes in Response to Plant Hormones

**FIGURE 7:**
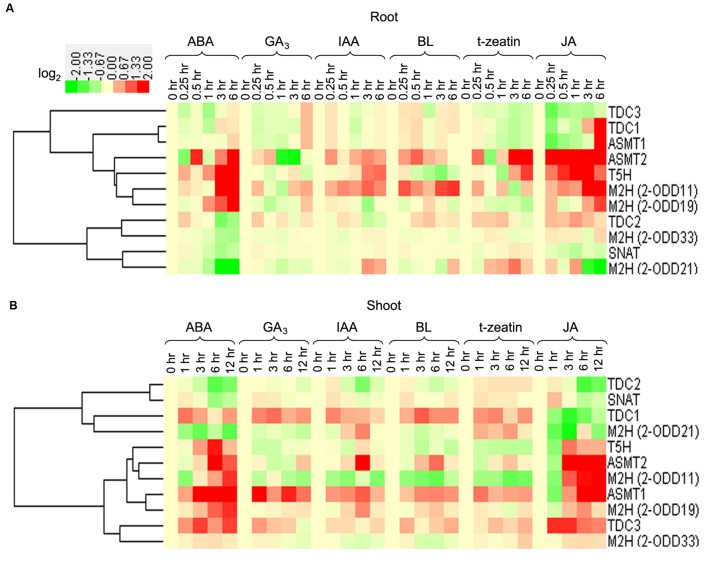
**The transcriptional profile of rice melatonin synthesis and catabolic genes in root (A) and shoot (B) in response to plant hormones.** The transcriptional expression data were obtained from RiceXPro (http://ricexpro.dna.affrc.go.jp/). The cluster analysis of expression profile for each gene in various tissues is shown as normalized data (log_2_) in relative to the 0 h of treatment which was set as 0.

In response to ABA and JA treatments, the transcripts of *OsT5H*, *OsTDC2*, -*3*, and *Os2-ODD19* displayed significantly up-regulation in root or shoot (**Figure 7**). The transcript of *Os2-ODD11* was significant up-regulated after IAA, BL, and JA treatments in root, but was strongly down-regulated after IAA, BL, and tZ treatments in shoot. The transcripts of *OsTDC1* and *OsASMT1* showed up-regulation after ABA, GA_3_, IAA, BL, and tZ treatments in shoot, while *OsTDC3* expression was induced after ABA, IAA, BL, tZ, and JA treatments in shoot (**Figure 7**).

Generally, melatonin-related genes showed different expression profiles in root or shoot tissues for the same treatment (**Figure 7**). The transcripts of *OsTDC1*, *-3*, *Os2-ODD33*, and *OsASMT1* were up-regulated in the shoots, but were not significantly regulated or down-regulated in the roots after ABA and tZ treatments. The expression of *Os2-ODD11* was increased in roots, but was decreased in shoots after ABA and BL treatments. Although some melatonin-related genes were from the same family, they exhibited different responses to plant hormones treatments, such as *OsTDC2* and *OsTDC3* in roots, *Os2-ODD11* and *Os2-ODD21* in roots, *OsASMT1* and *OsASMT2* in shoots. Thus, the transcriptional response of melatonin-related genes to plant hormones treatments in roots and shoots may provide new insight into crosstalk between melatonin and plant hormones, as well as mechanism underlying melatonin-mediated signaling in rice.

### Gene Expression Profile in Whole Leaf of Rice Melatonin Synthesis and Catabolic Genes Inoculated with Pathogen Infection

Because melatonin plays important roles in response to pathogen infection (Yin et al., 2013; Lee et al., 2014, 2015; Reiter et al., 2015; Shi et al., 2015a; Zhao et al., 2015), so we analyzed the expression profile of rice melatonin synthesis and catabolic genes in response to pathogen inoculation to identify the candidate genes for further analysis.

**FIGURE 8:**
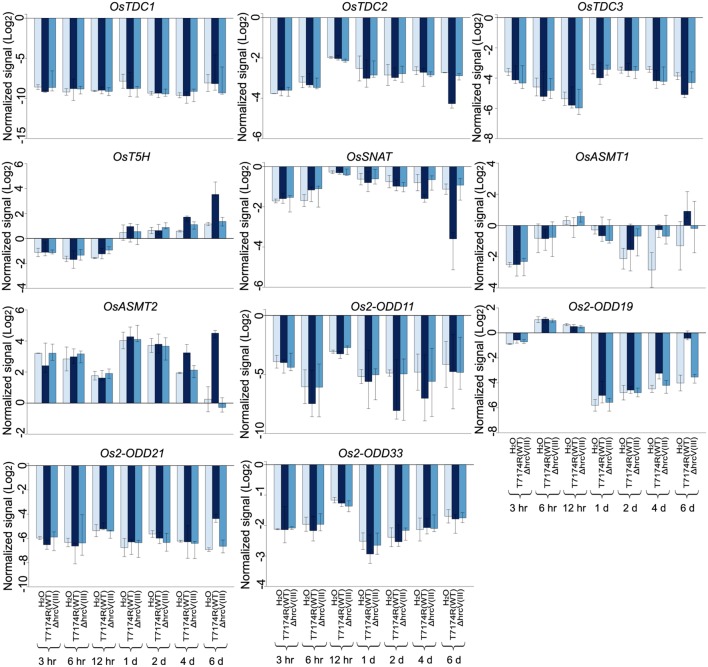
**Gene expression profile in whole leaf of rice melatonin synthesis and catabolic genes inoculated with *Xoo*, the causal agent of bacterial blight disease.** The transcriptional expression data were obtained from RiceXPro (http://ricexpro.dna.affrc.go.jp/). Rice leaves inoculated with T7114R, a wild-type strain, or 74HrcV::Km, *ahrcV* mutant deficient in type III secretion (T3S) system, were harvested at 3 hpi, 6 hpi, 12 hpi, 1 dpi, 2 dpi, 4 dpi, and 6 dpi. The expression profile for each gene in various tissues is shown as normalized data (log_2_).

After inoculation with *Xoo*, *OsASMT2* expression was induced during almost all the time-points, while *OsASMT1* transcript was decreased at these time points (**Figure 8**). The transcripts of *OsT5H* and *Os2-ODD19* were induced during 1 to 6 dpi and 6 to 12 hpi, respectively (**Figure 8**). *OsTDC1*, -*2*, -*3*, *Os2-ODD11*, -*21*,-*33*, and *OsSNAT*, expressions were decreased during all the treated time points (**Figure 8**). Interestingly, the transcripts of *OsASMT1* and *OsASMT2* were increased after 6 dpi of wild-type strain T7114R, but were decreased after 6 dpi of Δ*ahrcV(III)* strain. T3S is essential for *Xoo*T7174R conferred plant disease, and Δ*ahrcV(III)* resulted in less plant disease in rice leaves (Sato et al., 2011b, 2013). Thus, the results indicate the possible role of *OsASMT1* and *OsASMT2* in immune response to *Xoo*.

After inoculation with the blast fungus (*M. oryzae*), the transcript levels of three genes (*OsT5H*, *OsASMT1*, and *Os2-ODD11*) showed up-regulation at most treated time points, while those of four genes (*OsTDC1*, -*3*, *OsSNAT*, and *Os2-ODD21*) showed down-regulation (**Supplementary Figure S1**). In response to inoculation with the three strains of *M. oryzae* harboring *AVR-Pia* and *AVR-Pish* (Pia/Pish × P91-15B, Pish × P91-15B, and Pish × Kyu77-07A), the transcript levels of *OsASMT1*, *Os2-ODD11*, -*19*, -*33*, and *OsT5H* showed significant up-regulation, while those of *OsTDC3*, *OsSNAT*, and *Os2-ODD21* were seriously down-regulated at all time points (**Supplementary Figure S1**). *OsASMT2* expression showed no obvious trends in response to inoculation with the two former, while was induced at all the treated time-points after inoculation with Pish × Kyu77-07A strain (**Supplementary Figure S1**). In response to inoculation with the *pish* mutant of *M. oryzae* (Δ*Pish* × Kyu77-07A), the expressions of six genes (*OsTDC1*, *OsT5H*, *OsASMT1*, -*2*, *Os2-ODD11*, and *19*) were significantly induced at all time points, while four genes (*OsTDC2*, -*3*, *OsSNAT*, and *Os2-ODD33*) were seriously down-regulated (**Supplementary Figure S1**). *Os2-ODD21* expression was strongly repressed at 2 dpi. Notably, *Os2-ODD33* expression was induced at all the treated time-points after inoculation with the three strains of *M. oryzae* harboring *AVR-Pia* and *AVR-Pish*, but was repressed in response to inoculation with Δ*Pish* × Kyu77-07A strain (**Supplementary Figure S1**).

### The Transcriptional Profile of Rice Melatonin Synthesis and Catabolic Genes in Response to Abiotic Stress Treatments

**FIGURE 9:**
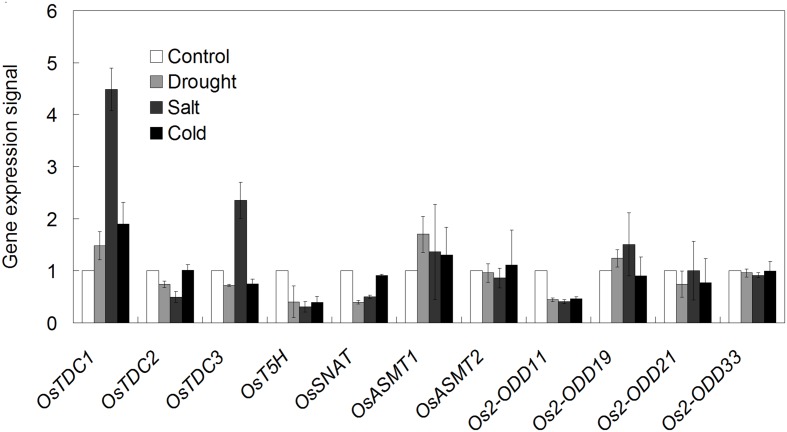
**The transcriptional profile of rice melatonin synthesis and catabolic genes in response to abiotic stress treatments.** The transcriptional expression data were obtained from Rice eFP Browser (http://bar.utoronto.ca/efprice/cgi-bin/efpWeb.cgi).

Melatonin is widely involved in plant stress responses (Shi et al., 2015b,d). Thus, investigation of the expression profiles of melatonin-related genes of rice in response to various abiotic stresses is needed. After drought treatment, the transcripts of *OsTDC1*, *OsASMT1*, and *Os2-ODD19* were found to be up-regulated between 1.2 and 1.8-folds, whereas those of *OsTDC2*, -*3*, *Os2-ODD11*, -*21*, *OsT5H*, and *OsSNAT* were strongly repressed in comparison to the control (**Figure 9**). After salt treatment, the transcripts of *OsTDC1*, -*3*, *OsASMT1*, and *Os2-ODD19* were increased between 1.3 and 4.5-fold, whereas NaCl strongly repressed *OsTDC2*, *OsT5H*, *OsSNAT*, and *Os2-ODD11* expressions (**Figure 9**). After 4°C treatment, *OsTDC1* and *OsASMT1* transcript levels were slightly increased, whereas the expressions of six genes (*Os2-ODD11*, -*19*, -*21, OsTDC3*, *OsT5H*, and *OsSNAT*) were obviously down-regulated (**Figure 9**). Notably, the transcript of *OsTDC3* was significantly increased after salt treatment, but was seriously decreased in response to drought and cold treatments (**Figure 9**). The result indicated that *OsTDC1* and *OsTDC3* may be involved in salt stress response.

## Discussion

To our knowledge, this is the first study systematically analyzed the diurnal and circadian transcriptional profile of melatonin synthesis and catabolic genes throughout the entire growth stages in rice. Our study confirmed that *OsTDCs* showed higher expression level at reproductive 1 and reproductive ripening stages then other stages (**Figure 5**). In cherry fruit (*Prunus avium*), the expression level of *PaTDC* was positively correlated to melatonin concentration throughout the entire period, and showed regularly circadian rhythm during a 24 h period with two peaks at 5:00 and 14:00 (Zhao et al., 2013), indicating that the melatonin concentration was higher in that two stages then others. The expression pattern of *OsTDC3* also showed circadian rhythm on 16 DAT (**Figure 6**). Similarly, this expression pattern was also existed at *Os2-ODD19* during the entire development (**Figure 5**). These results suggested that *OsTDC3* and *Os2-ODD19* may be involved in modulating endogenous during the entire development in rice.

Melatonin is widely involved in plant growth and development, as well as stress responses (Bajwa et al., 2014; Meng et al., 2014; Wang et al., 2014; Zuo et al., 2014; Liang et al., 2015; Shi et al., 2015b). In apple (*Malus prunifolia*), the transcripts of melatonin synthesis genes (*MdTDC1*, *MdAANAT2*, *MdT5H4*, and *MdASMT1*) was induced after drought treatment (Li et al., 2014). The concentration of melatonin was increased in barley roots and lupin after cold, drought and salt treatments (Arnao and Hernández-Ruiz, 2009, 2013). Consistently, *OsTDC1* and *OsASMT1* transcript levels were increased after drought, salt and cold treatments (**Figure 9**), indicating their possible involvement in abiotic stress response.

Recently, melatonin was shown to function as positive modulator against plant pathogen infection (Yin et al., 2013; Lee et al., 2014, 2015; Reiter et al., 2015; Shi et al., 2015a; Zhao et al., 2015). Exogenous application of melatonin improved apple resistance to *D. mali*, the pathogen of Marssonina apple blotch (Yin et al., 2013), enhanced *Arabidopsis* and tobacco defense against *P. syringae* pv. tomato DC3000 (Lee et al., 2014). Moreover, *Arabidopsis snat* knockout mutants exhibited increased susceptibility to the avirulent pathogen *P. syringae* pv. tomato DC3000 with decreased SA levels and reduced defense genes expression compared with wild-type (Lee et al., 2015). However, whether *OsSNAT* also involves in the regulation of plant immunity remains unknown. Treatments with melatonin significantly enhanced antioxidant protection in rice, suggesting that melatonin plays a major role in regulating pathogen infection (Liang et al., 2015). In response to bacterial pathogen infection, some genes showed similar expression profiles (**Figure 8**). The differential response of melatonin-related genes to pathogen infection in different kinds implied the dual mechanisms underlying melatonin-related genes mediated pathogens responses.

Previous studies have revealed that melatonin had significant effect in regulating ABA and GA_4_ in plant response to salinity and drought stress (Li et al., 2014; Zhang H.J. et al., 2014). Additionally, melatonin shared the common substrate (tryptophan) with IAA (Arnao and Hernández-Ruiz, 2014), and AXR3/IAA17 is involved in *Arabidopsis* melatonin signaling underlying senescence (Shi et al., 2015c). Thus, genome-wide expression analysis of melatonin-related genes in response to plant hormones may provide new insight into crosstalk between melatonin and plant hormones. Plant hormones, such as ABA, SA, and GA, related with most of the plant physiological responses, including water logging, drought and salt stress responses (Yang et al., 2004; Kim et al., 2011, 2014; Shimamura et al., 2014). Melatonin is also a regulator of ABA and GA_4_ in plant response to salinity and drought stress (Li et al., 2014; Zhang H.J. et al., 2014). Moreover, the expression of *OsASMT2* and *OsASMT3* were induced after ABA and JA treatments at 1-month-old detached rice leaves, while were down-regulated in response to ethephone, zeatin, and SA stress. *OsASMT1* also showed up-regulation upon ABA and JA stress treatment, but did not display obvious trends during ethephone, zeatin, and SA treatments (Park et al., 2013a). In this study, the transcripts of four genes (*Os2-ODD11*,-*19, OsASMT2*, and *OsT5H*) and five genes (*OsASMT1*, -*2*, *OsT5H*, *Os2-ODD19*, and *OsTDC3*) were increased in response to ABA stress during 3 h to 6 h treatment in root and during 3 to 12 h in shoot, respectively. Under JA stress, the transcripts of three genes (*OsASMT2*, *OsT5H*, and *Os2-ODD11*) and three genes (*OsASMT1*, -*2*, and *Os2-ODD11*) were significantly increased during 0.25 to 6 h in root and 3 h to 12 h in shoot, respectively. Thus, different transcriptional responses of melatonin-related genes in hormone specific manners, suggested the dual role and crosstalk between melatonin and various hormones.

It is widely known that ABA is the most important regulator of abiotic stress (Kim et al., 2011, 2014; Shimamura et al., 2014), and JA serves as the major defense hormone that are associated with pathogen infection (Van der Ent et al., 2009; Ballaré, 2011; Xie et al., 2011; Yamada et al., 2012; Yang et al., 2013; Campos et al., 2014). More recently, the crucial role of ABA in virulence of rice blast fungus *M. oryzae* is confirmed (Spence et al., 2015), the involvement of JA in abiotic stress response is also largely confirmed (Riemann et al., 2015; Wasternack and Strnad, 2016). We highlight the commonly regulation of rice melatonin-related genes by ABA, JA, pathogen infection and various abiotic stresses (**Figures 7–9**), indicating the possible role of these genes in multiple stress responses and underlying crosstalks of plant hormones, especially ABA and JA. Weeda et al. (2014) identified 1308 differentially expressed genes (566 up-regulated genes and 742 down-regulated genes) exhibiting at least of twofold change by exogenous melatonin treatment in *Arabidopsis*, and many of them are enriched in plant hormone signaling. These differentially expressed genes include 52 genes in auxin signaling, 50 genes in ABA signaling, 67 genes in JA pathway, and 42 genes in ET pathway. Our studies together with the data of Weeda et al. (2014) further indicate the interaction among melatonin, ABA and JA pathways.

We have to pointed out the possible limitation of this study, since the different changes in expression levels of the various genes do not always explain in a simple way why melatonin concentrations increase or decrease under the different conditions. On one hand, there may be difference between expression level and enzyme activity, such as the posttranslational regulation of AANAT in primates via phosphorylation/dephosphorylation and association/dissociation of a 14-3-3 protein, which is decisive for the melatonin rhythm in those organisms (Ganguly et al., 2001, 2005). On the other hand, incomplete knowledge of rate-limiting enzymes or isoenzymes may also lead to the difference. Further studies by other methods may give more clues.

Taken together, the expression patterns of 11 melatonin related genes from rice were synthetically analyzed at different periods and after different treatments in this study. These information may provide abundant resources for functional characterization of melatonin related genes. The differential expression patterns of melatonin related genes in different tissues throughout entire growth stages and stress responses will be useful to investigate *in vivo* role of specific gene in rice development and circadian rhythm. Thus, this study will contribute to better understand the melatonin biosynthesis and catabolic pathway as well as their association with development and stress responses in rice. Further functional analysis of identified candidate genes with potential involvement in circadian rhythm and stress responses will give shed more lights in melatonin-mediated signaling as well as underlying molecular mechanism.

## Author Contributions

HS conceived and directed this study, analyzed the data, wrote, and revised the manuscript; YW and HZ performed the experiments, analyzed the data, wrote, and revised the manuscript; WH and LC analyzed the data and revised the manuscript; CH provided suggestions and revised the manuscript. All authors approved the manuscript and the version to be published, and agreed to be accountable for all aspects of the work in ensuring that questions related to the accuracy or integrity of any part of the work are appropriately investigated and resolved.

## Conflict of Interest Statement

The authors declare that the research was conducted in the absence of any commercial or financial relationships that could be construed as a potential conflict of interest.

## Footnotes

1 http://ricexpro.dna.affrc.go.jp/

2 http://bar.utoronto.ca/efprice/cgi-bin/efpWeb.cgi

3 http://bonsai.hgc.jp/~mdehoon/software/cluster/software.htm

4 http://jtreeview.sourceforge.net/

## Supplementary Material

Table S1 **The detailed data used for Figures 3–7 and Figure 9.** The original data were obtained from RiceXPro (http://ricexpro.dna.affrc.go.jp/) and Rice eFP Browser (http://bar.utoronto.ca/efprice/cgi-bin/efpWeb.cgi).

FIGURE S1 **Gene expression profile in whole leaf of rice melatonin synthetic and degradated genes inoculated with the blast fungus, *M. oryzae*.** The transcriptional expression data were obtained from RiceXPro (http://ricexpro.dna.affrc.go.jp/). Three lines of rice cultivar Nipponbare carrying the blast resistance genes (*Pia*, *Pish*) were inoculated with two strains of *M. oryzae* harboring *AVR-Pia* and *AVR-Pish*. Rice seedlings at the 4-leaf stage were inoculated with the conidial suspension of *M. oryzae* and the leaves were harvested at 1, 2, 3, and 5 dpi. The expression profile for each gene in various tissues is shown as normalized data (log_2_).

## ABBREVIATIONS

2-ODD: 2-oxoglutarate-dependent dioxygenase
AANAT: arylalkylamine *N*-acetyltransferase
ABA: abscisic acid
ASMT: *N*-aceylserotonin methyltransferase
AXR3: Auxin Resistant 3
BL: indole-3-acetic acid
DAT: days after transplanting
GA: gibberellic acid
GEO: Gene Expression Omnibus
hpi: hour post inoculation
IAA: indole-3-acetic acid
JA: jasmonic acid
M2H: melatonin 2-hydroxylase
SNAT: serotonin *N*-acetyltransferase
T3S: type III secretion system
T5H: tryptamine 5-hydroxylase
TDC: tryptophan decarboxylase
tZ: trans-zeatin
*Xoo*: *Xanthomonas oryzae* pv. *oryzae*.
