# Supplementary figures and images for: Comparative Transcriptional Profiling of Melatonin Synthesis and Catabolic Genes Indicates the Possible Role of Melatonin in Developmental and Stress Responses in Rice
Source: Front Plant Sci. 2016 May 18;7:676. doi: 10.3389/fpls.2016.00676 (PMC4870392; doi:10.3389/fpls.2016.00676)

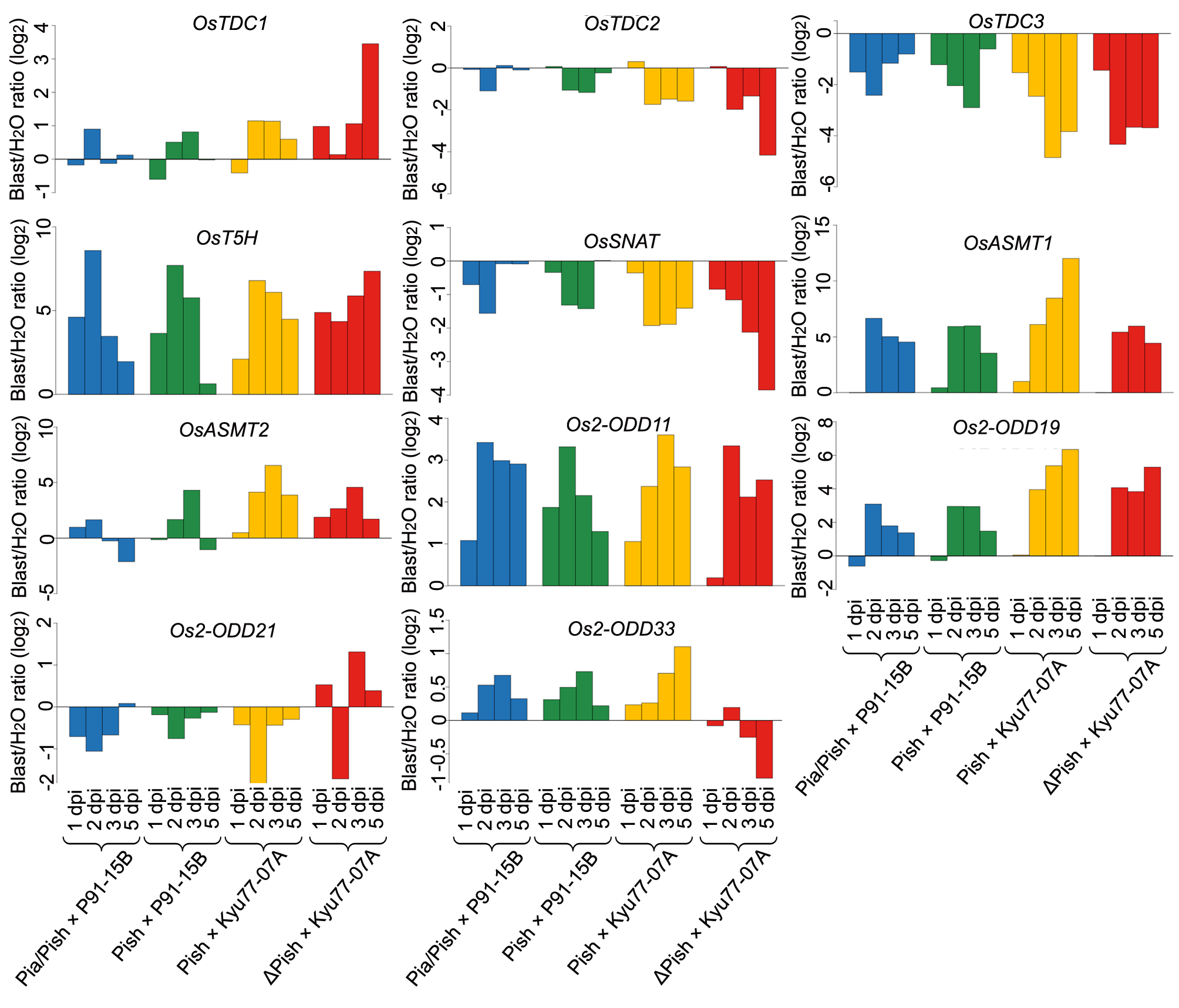

Supplement: Table S1 — The detailed data used for Figures 3–7 and Figure 9. The original data were obtained from RiceXPro (http://ricexpro.dna.affrc.go.jp/) and Rice eFP Browser (http://bar.utoronto.ca/efprice/cgi-bin/efpWeb.cgi). [file Image_1.TIF]
